# Antioxidants in Animal Feed

**DOI:** 10.3390/antiox11091760

**Published:** 2022-09-06

**Authors:** Jie Wang, Wei Si, Zhenyu Du, Junmin Zhang, Min Xue

**Affiliations:** 1 National Aquafeed Safety Assessment Center, Institute of Feed Research, Chinese Academy of Agricultural Sciences, Beijing 100081, China; 2 State Key Laboratory of Animal Nutrition, Institute of Animal Sciences of Chinese Academy of Agricultural Sciences, Beijing 100193, China; 3 School of Life Sciences, East China Normal University, Shanghai 200241, China

Production animals are often exposed to several oxidative stress conditions, including, but not limited to, heavy metals, alternative protein sources, environmental stress, disease, high densities, as well as handling, which may suppress growth performance, animal health and production, subsequently impacting economic feasibility. Promising research results have revealed that the administration of natural or synthetic antioxidants in the diet would be an important nutritional strategy to mitigate the negative influence induced by oxidative stress conditions. The Special Issue “Antioxidants in animal feed” has been conceived to set out the knowledge on the effects of dietary antioxidants on host health and performance of production animals, including livestock, poultry and fish. It provides various nutritional approaches to improve antioxidant capacity and benefit host health in animal production. Here, we offer an overview of the contents of this Special Issue, which collects 17 original articles.

For livestock and poultry, oxidative stress could affect ovarian function. Wang et al. found that oxidative stress could decrease the laying performance, ovarian function and influence gut microbiota and body metabolites in the layer model [1]. They then explored the role of melatonin on ovary oxidative stress, suggesting melatonin could exert an amelioration in ovary oxidative stress through the SIRT1-P53/FoxO1 pathway. Melatonin is considered as a bio-antioxidant. Peng et al. evaluated the impacts of dietary melatonin supplementation during pregnancy on reproductive performance, maternal–placental–fetal redox status, placental inflammatory response and mitochondrial function [2]. They concluded that melatonin supplementation during gestation could improve maternal–placental–fetal redox status and reproductive performance by ameliorating placental antioxidant status, inflammatory response and mitochondrial dysfunction. The work from Xu et al. focused on the potential effects of adding acidifiers to drinking water [3]. The results showed that supplementing drinking water with an acidifier has potential as an antioxidant, which was reflected in improvements in growth performance, immunity, antioxidant capacity and intestinal flora. The study by Liu et al. determined the effects and mechanisms of increased consumption of methionine by sows and piglets on the capacity of the progeny to counteract lipopolysaccharide (LPS) challenge-induced injury in the liver and spleen of piglets [4]. The results showed that dietary methionine supplementation alleviated liver and spleen damage that was induced by the LPS challenge. In addition, the results indicated that beneficial effects of dietary methionine were potentially due to the increased antioxidant capacity and inhibition of the TLR4 and NOD signaling pathway.

Various studies focused on the use of antioxidants in ruminates to improve health, performance and product quality. Wang et al. explored the effects of L-glutamine (L-Gln) on calves during weaning [5]. They found that a dietary lower-level L-Gln supplementation (1 and 2% of DMI) had higher average daily gain, glutathione peroxidase and IgG concentration and villus height/crypt depth of the duodenum and jejunum, as well as lower cortisol, haptoglobin and interleukin-8 concentration in weaned calves. These results provided evidence that the addition of L-Gln in the diet improved the negative effects of sudden weaning in calves. A study from Ma et al. investigated the effects of rumen-protected glucose (RPG) on the hepatic oxidative/antioxidative status and protein profile [6]. They showed that RPG supplementation reduced insulin sensitivity but increased the liver triglyceride concentration and the oxidative stress in early postpartum cows, which may indicate an increased risk of liver metabolic disorders caused by RPG supplementation. Wang et al. found that a plant-protein-based milk replacer had a negative effect on calves’ liver function, immunity and antioxidant capacity [7]. In addition, transcriptome analysis revealed that energy metabolism, immune function and mineral metabolism showed differences during the pre-weaning period, while during the post-weaning period, osteoclast differentiation and metabolic pathways showed a difference. Kong et al. studied the potential of *Acremonium terricola* culture (ATC) of ATC as a new feed additive in dairy cow feeding [8]. The results showed that ATC improved milk yield and milk protein yield. Furthermore, the improvement in milk yield was likely related to improved immune function and antioxidant capacity.

Regarding fish production, various studies show how to mitigate the oxidative stress caused by unconditional production conditions using micronutrients. In particular, Wu et al. explored the effects of vitamin A on the muscle quality, nutritional quality and antioxidative ability of grass carp [9]. The results highlighted that dietary Vitamin A could improve flesh quality by increasing antioxidant capacity via the Nrf2/Keap1 signaling pathway. Similarly, dietary vitamin C can attenuate oxidative damage, inflammation and acute hypoxia-induced apoptosis in gibel carp via the Nrf2/Keap1 signaling pathway [10]. These findings further suggest that vitamins A and C, as essential micronutrients, could be powerful antioxidants in the diet to regulate antioxidant capacity via certain potential signaling pathways, such as Nrf2/Keap1. Further, the work of Xu et al. focused on docosahexaenoic acid (DHA), as a nutritional modulator, to alleviate palmitic-acid-induced inflammation of macrophages via the TLR22-MAPK-PPAR/Nrf2 pathway in large yellow croakers, thereby improving the utilization rate of palm oil in aquafeed [11]. Another study found that dietary glutamine could regulate immune and antioxidant capacity to protect against *Flavobacterium columnare* infection in yellow catfish [12]. The authors suggest their study firstly demonstrated the regulatory roles of glutamine in the fish immune and antioxidant system and reported its inhibitory effects on fish apoptosis and autophagy during pathogenic infection. Furthermore, Shi et al. found that oxidized-fish-oil diets can cause negative physiological health effects in channel catfish, while adding taurine can increase growth and antioxidant ability, reduce lipid deposition and improve intestinal health [13]. These findings could advance the understanding of the molecular mechanism of oxidative stress and provide nutritional mitigative strategies via supplemented micronutrients in aquaculture production.

Special attention has also been paid to plant extracts, functional components or alternative protein sources. Starch is necessary as a binder and sweller during extrusion processing of pelleted aquatic feeds. However, carnivorous fish, for example, largemouth bass, fed excess starch may induce metabolic liver disease [14]. Liang et al. found that largemouth bass fed high-starch feed induced oxidative stress and lipid metabolic disorder, while dietary olive extract could improve antioxidant capacity, anti-inflammatory responses and lipid metabolism, but could not completely repair high-starch-diet-induced lipid metabolic disorder [15]. Xu et al. also reported that probiotic *Lactobacillus plantarum* MR1 ameliorated high-carbohydrate-diet-induced hepatic lipid accumulation and oxidative stress by increasing the circulating uridine [16]. Additionally, conventional soybean meal, replacing fishmeal protein in aquatic feed, could adversely influence the growth performance and health of the host. Wang et al. showed that the mixture of plant extracts (thymol and carvacrol) and chelated trace elements (Cu, Mn and Zn) in the diet could mitigate soymeal-induced adverse effects on growth and disease resistance through the improvement in antioxidant capacity and regulation of gut microbiota [17]. Interestingly, *Arthrospira platensis*, a blue-green alga, could activate the antioxidant response and alleviate oxidative stress and pigmentation disorder induced by air exposure in yellow catfish [18].

All the research articles in this Special Issue show that establishing a better understanding of oxidative stress is of pivotal importance in production animals. The variety of subjects treated proves that this is a complex and multifaceted topic, on which researchers are working from different viewpoints and perspectives. We thank all the authors for their contributions. We hope that this Special Issue will encourage more scientists to move forward on the path to increasing knowledge on the effect of natural or synthetic antioxidants on the growth and health of production animals.
